# Recurrent acute appendicitis after recovery from scrub typhus that was associated with hemophagocytic lymphohistiocytosis and other severe complications in children: a case report

**DOI:** 10.3389/fmed.2025.1528903

**Published:** 2025-05-29

**Authors:** Min Yang, Yang Wang, Li-Li Luo, Li-Na Qiao

**Affiliations:** ^1^Department of Pediatrics, West China Second University Hospital, Sichuan University, Chengdu, China; ^2^Key Laboratory of Birth Defects and Related Diseases of Women and Children of Sichuan University, Ministry of Education, Chengdu, Sichuan, China; ^3^NHC Key Laboratory of Chronobiology, Sichuan University, Chengdu, China

**Keywords:** acute appendicitis, scrub typhus, hemophagocytic lymphohistiocytosis, complication, children

## Abstract

**Background:**

With the advancement of diagnostic technology, diagnosing and treating scrub typhus (ST) has become easier, and ST is currently commonly associated with various complications. At present, there is no report of a recurrent acute appendicitis requiring surgical resection after the successful treatment of ST with severe complications, such as hemophagocytic lymphohistiocytosis (HLH), in children during follow-up.

**Case presentation:**

We report the case of a 10-year-old girl from Sichuan, China, who had fever, abdominal pain, and lethargy. Abdominal computed tomography (CT) scans indicated appendicitis, and the surgeons indicated that surgery was unnecessary. She was then admitted to the Pediatric Intensive Care Unit (PICU) and rapidly developed severe complications (HLH, septic shock, acute kidney injury, acute respiratory distress syndrome, and disseminated intravascular coagulation) within 24 h after admission. She was diagnosed with ST by metagenomics next-generation sequencing (mNGS). After treatment with doxycycline, dexamethasone (DEX), and etoposide, as well as advanced life support, she recovered after 25 days of hospitalization and was discharged. However, she underwent a laparoscopic appendectomy due to abdominal pain a month after a reduction in the dose of DEX. The appendix was 6 cm long and 1.0 cm in diameter, and the pathological report suggested simple acute appendicitis. After 11 months of follow-up, that is, 10 months after the appendectomy, all indicators were normal and no similar abdominal pain recurred.

**Conclusion:**

Pediatricians should be vigilant and should initiate HLH treatment protocols when ST-associated HLH occurs with other severe complications. Acute appendicitis may not only occur during the course of ST, but may also occur after successful treatment for ST.

## Introduction

Scrub typhus (ST) is a disease (1) that occasionally occurs after the bite of mite larvae infected by *Orientia tsutsugamushi* (a Gram-negative bacteria). Studies have summarized the common clinical manifestations of ST in Nepal (for the 23 years from 2000 to 2023) (2) and in China (for the 72 years from 1950 to 2022) (3), which include fever, headache, cough, and abdominal pain. ST has attracted attention because it may induce an overreaction of the human immune system, produce a cytokine storm, cause damage to important organs, and cause serious or even fatal complications (4). Kaushik Mukhopadhyay et al. (4) analyzed the literature on the complications of pediatric ST reported in the past 30 years (1990–2020) and proposed that multiple organ dysfunction syndrome (MODS) was the main cause of death. MODS is a disease (5) of two or more organ systems (cardiovascular, respiratory, renal, hepatic, neurological, and hematologic system alterations) arising from dysfunction from any cause that requires intervention to maintain homeostasis. Hemophagocytic lymphohistiocytosis (HLH) is a rare, life-threatening condition that is induced by ST after overactivation of the systemic immune system (6). Most cases recovered after anti-rickettsial therapy, with/without steroid therapy and advanced life support (2, 6–9).

We report the case of a child initially diagnosed with acute appendicitis who was subsequently confirmed to have ST, MODS, and HLH. The child was discharged after anti-rickettsial therapy, dexamethasone (DEX), etoposide, and advanced life support. The reoccurrence of acute appendicitis after reduction of the DEX dose has not been reported so far.

## Case presentation

A previously healthy 10-year-old girl presented with abdominal pain, fever, and lethargy of 1 week, 6 days, and 5 days duration, respectively. The abdominal pain was paroxysmal and colicky, was at the right lower abdomen, and was relieved in the flexed position; it was associated with non-projectile vomiting of stomach contents 1–2 times per day. There was also remittent fever, which was accompanied with chills, lethargy, and a dull bilateral headache. The dorsum of the left foot was swollen, painful, red, and hot, but there was no diarrhea or bleeding from the skin or mucous membranes. Before being admitted to the Pediatric Intensive Care Unit (PICU), she had visited the local hospital several times. After treatment with oral and intravenous cephalosporin antibiotics, the swelling, pain, redness, and hotness of the dorsum of the left foot subsided, and she was admitted to the PICU because of the persistent fever, abdominal pain, and lethargy. One week before symptom onset, there was a stone scratch on the back of her left foot. Mosquito bite history was denied, and there was no similar discomfort in other family members. She lives in the rural Xichang City, Sichuan Province, China.

Physical examination revealed a poor mental state, drowsiness, increased respiration, generalized petechiae, a scab on the dorsum of the left foot, no traces of eschar on the skin of the entire body, and no enlargement of superficial lymph nodes. She also had abdominal distension and tenderness with no rebound pain. The liver and spleen were not palpable, and the capillary refill time was 3 s.

Laboratory tests indicated pancytopenia (neutrophil count 0.2 × 10^9^/L, hemoglobin 65 g/L, platelet count 7 × 10^9^/L), increased hypersensitive C-reactive protein (Hs-CRP) levels (124.4 mg/L), increased procalcitonin (PCT) levels (5.12 ng/mL), elevated liver enzymes (alanine transaminase [ALT] 264 U/L and aspartate aminotransferase [AST] 352 U/L), decreased albumin (ALB) levels (24.7 g/L), hypertriglyceridemia (3.26 mmol/L), hyperferritinemia (8504.30 ug/L), significantly increased lactate dehydrogenase (LDH) levels (1,176 U/L; normal values 120–246 U/L), and elevated cytokines (significantly abnormal interleukin [IL] 2 receptor [IL-2R] levels 8367.2 U/mL; IL-6 levels 112.89 pg./mL, IL-8 levels 208.25 pg./mL, IL-10 levels 229.38 pg./mL, and tumor necrosis factor alpha [TNF-*α*] levels 221.77 pg./mL). Screening for diffuse intravascular coagulation revealed abnormalities (the prothrombin time was 17.4 s, the activated partial thromboplastin time was 67.5 s, the international normalized ratio was 1.6, the fibrinogen level was 129 mg/dL, the D-dimer level was 16.57 mg/L, the fibrinogen degradation product level was 37.29 ug/mL, and the antithrombin III level was 38%). The serum creatinine level increased (102 μmol/L), and bone marrow examination revealed the phagocytosis of histiocytes. A cranial computed tomography (CT) scan showed no abnormality. CT of the chest and abdomen indicated inflammation of both lungs, partial consolidation and atelectasis of the lower lobe of the left lung, a small pericardial effusion, a small pleural effusion bilaterally, liver and spleen enlargement, mild hepatic lymphatic stasis, fullness of the left adrenal gland, peritoneal thickening, abdomino-pelvic effusion (suggesting peritonitis), multiple small lymph nodes on the abdominal aorta and mesentery, a partially enlarged appendix, no swelling of the intestinal wall, and clear surrounding fat space. Metagenomics next-generation sequencing (mNGS) of her blood showed *Orientia tsutsugamushi* (high confidence; number of specific sequences 11,387; relative abundance 99.65%). The standard quantitative polymerase chain reactionq (qPCR) results confirmed the results of the mNGS analysis, with a cycle threshold (Ct) of 33.96. Three blood cultures were negative.

Before admission, she was surgically evaluated because of the fever, abdominal pain, abdominal distension, and abdominal tenderness, and the abdominal CT was suggestive of peritonitis and an enlarged appendix but was deemed not to require surgery.

After admission, she was immediately administered high-flow nasal cannula (HFNC)-assisted ventilation and meropenem combined with linezolid as antibiotic therapy. Blood products were administered to treat the disseminated intravascular coagulation (DIC), and DEX (10 mg/m^2^/day) was administered for 2 weeks, with its dose being reduced by half every 2 weeks for 6 weeks subsequently. The DEX was administered intravenously (i.v., guttae [i.v.gtt]) and combined with etoposide (150 mg/m^2^ twice a week for 1 week, followed by 1 week of discontinuation due to neutrophil deficiency, then 150 mg/m^2^ twice a week again for 1 week, and one dose of 75 mg/m^2^; a total of 5 doses) to treat the HLH.

Within 24 h after admission, she developed shock and acute respiratory distress syndrome (ARDS), which manifested as rapid respiration (respiratory rate of 56 times/min) despite HFNC-assisted ventilation, decreased blood pressure (BP 64/33 mmHg), systemic edema, and significantly reduced urine volume (315 mL, equivalent to 0.39 mL/kg/h). The three concave sign was positive, and rales were absent in both lungs. Airway intubation and invasive ventilator-assisted ventilation, dilatation, and vasoactive drugs (i.v.gtt norepinephrine 0.2 ug/kg/min for 6 days and i.v.gtt dobutamine 5 ug/kg/min for 3 days) were used to improve circulation. Continuous renal replacement therapy (CRRT, 75 h), the body temperature remained high, the entire abdomen was still tense, the infection monitoring indices increased, metronidazole was added to the antibiotic therapy on the third day, and other treatments were also administered.

On the 5th day of admission, the mNGS of the blood showed *Orientia tsutsugamushi* (high confidence, specific sequence number 11387, relative abundance 99.65%). Linezolid was discontinued, and doxycycline (2 mg/kg, twice a day) was added to her treatment.

On the 8th day of admission, the tracheal catheter was removed after the patient passed the withdrawal test.

On the 15th day after admission, the platelet count improved to more than 100 × 10^9^/L, and liver and kidney function test results, DIC screening results, and infection indicators returned to normal. Furthermore, the ferritin level reduced to 737.80 ng/mL, a mNGS of blood (sampled on the 14th day after admission and reported on the 15th day) showed no *Orientia tsutsugamushi*, and the neutrophil deficiency improved (neutrophils increased from 0.2 × 10^9^/L to 1.12 × 10^9^/L); however, the ferritin levels fluctuated (from 737.80 ng/mL to 927.30 ng/mL), and the ferritin level decreased to 144.40 ng/mL after 2 weeks of continued administration of three doses of etoposide, as described above.

The child’s temperature was normal for 10 days, and she improved and was discharged after 25 days of hospitalization.

**Figure 1:**
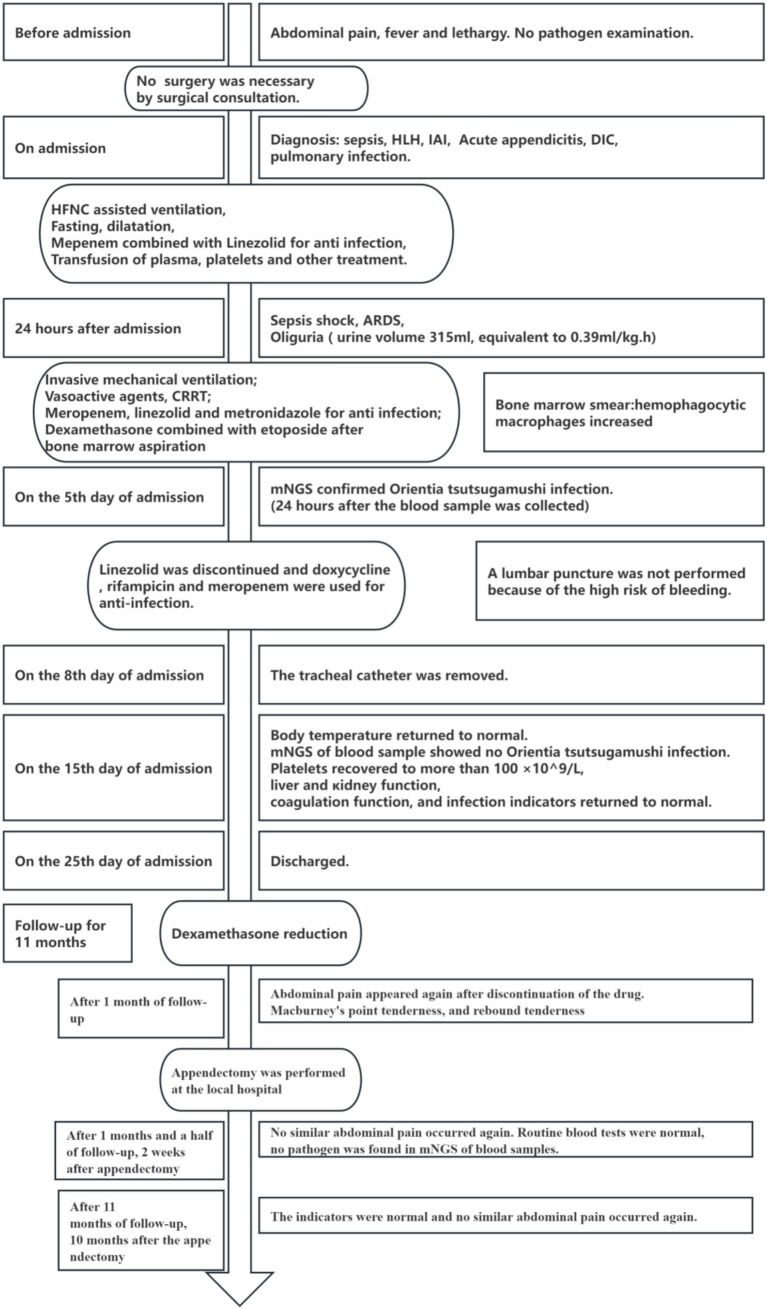
The timeline of disease progression and treatment.

She underwent a laparoscopic appendectomy due to abdominal pain, McBurney’s point tenderness, and rebound tenderness after discontinuation of DEX 1 month after discharge. The appendix was 6.0 cm long and 1.0 cm in diameter, and the pathological report suggested simple acute appendicitis. At the 11th month follow-up, 10 months after the appendectomy, the indicators, HLH-related markers (such as CD25) included, were normal and no similar abdominal pain reoccurred. The timeline of the disease progression and treatment is summarized in Figure 1.

## Discussion

Scrub typhus (ST) is distributed in the entire Pacific Rim of Asia (10), including but not limited to China. Since ST was included as one of the notifiable infectious diseases in China, surveillance has shown that it has become an increasingly serious public health problem (11) in Southwest China. As one of the main endemic areas in the Sichuan Province, the incidence of ST in Southwest China shows an increasing trend (12).

The clinical manifestations of ST are similar to those of other acute febrile diseases, and the manifestations of ST are diverse, affecting various organ systems (11, 12). Some articles have reported that abdominal pain is one of the main manifestations of ST but did not mention it after antibiotic treatment (2, 3). Only a few studies have mentioned surgery for suspected acute appendicitis or acute peritonitis of unknown surgical cause (13–15). Of these adults with ST who underwent surgery, only two had stomach perforation (14); in the other three, surgery confirmed the absence of a surgical cause (13, 15). To date, there are no reports of surgery in children with ST when acute appendicitis is considered. In our case, acute appendicitis was confirmed using the Alvarado score (nausea or vomiting, anorexia, pain in the right lower quadrant, and a body temperature of > 37.5°C) and an abdominal CT scan (16), and no surgical treatment was performed after surgical evaluation at the beginning of the disease. However, after DEX was discontinued as planned, the abdominal pain became evident again, and laparoscopic appendectomy was performed, which confirmed the appendicitis. For 9 months after the appendectomy, no similar abdominal pain has occurred.

The population of children with ST-associated HLH has been increasingly recognized (17), but the mortality rate (6.3–33.3%) is high when it is complicated by severe MODS in children with ST and HLH (6, 17–23). In our case, the patient suffered from ST, HLH, MODS (respiratory, renal, and hematologic system alterations), and even Multiple organ failure (MOF) (septic shock, ARDS, AKI, and DIC). In a retrospective analysis of 117 pediatric cases in 19 articles on ST-associated HLH (Table 1), 100 cases (85.5%) had complications other than HLH, which mainly involved the respiratory, circulatory, coagulation, and nervous systems; patients treated with steroids and/or etoposide and/or cyclosporine mainly had respiratory and nervous system involvement and/or MODS/MOF. Sixty-four patients (54.7%) were treated with steroids, 7 (6.0%) of whom had HLH, and whether the others had HLH was uncertain. Six (5.1%) patients with HLH were treated with etoposide, and 2 (1.7%) with cyclosporine. However, the above cases support the recommendation that patients with ST-associated HLH be treated with the recommended therapy for ST rather than conventional immunosuppressive treatment for ST-associated HLH (5, 17). In our case, we did not perform the Weil Felix test or test for positive IgM antibodies, but we sent the patient’s blood for mNGS as quickly as possible on a working day when mNGS could be performed and confirmed the diagnosis of ST (24). HLH was diagnosed using the 2004 HLH criteria (25), and five out of the eight criteria were present in our case, which is sufficient for diagnosing HLH (fever, cytopenia, hypertriglyceridemia, hyperferritinemia, emophagocytosis, and an IL-2R level > 2,400 U/mL). ST was treated, and the difference between our case and the cases in the 19 articles above was that we elaborated on DEX and etoposide therapy, as described in the case presentation section, which was administered according to the clinical response (fever, spleen, platelets, fibrinogen, and ferritin levels) and discontinued when HLH became inactive or resolved (normalization of fever, splenomegaly, cytopenia, triglyceride level, ferritin level, IL-2R level, and cerebrospinal fluid if the children had abnormal cerebrospinal fluid at the beginning of the illness) (26).

**Table 1:** Published articles on pediatric cases of scrub typhus with HLH.

Author	Country/year	Type of article	Male/female	Age	Illness days prior to admission	Days to confirm HLH	Complications other than HLH	Antibiotics for scrub typhus	Possible treatment for HLH (*n*, course of treatment)	In Hospital mortality	Cause of death	Follow-up time
Jayakrishnan MP, et al. (30)	India, 2011	Case	0/1	5 years	6 days	UD	Shock	Doxycycline	None	0	-	UD
Han DK, et al. (31)	Korea, 2012	Case	0/1	9 years	7 days	12 days	Encephalomyelitis	Roxithromyxin (7 days), doxycycline (2 weeks)	HLH-2004 protocol (1, 4 weeks and 7 weeks of induction therapy)	0	-	3 years (Permanent neurological sequelae)
Kwon HJ, et al. (32)	Korea, 2013	Case	1/0	8 months	10 days	10 days	Pulmonary hemorrhage, ARDS, seizure, DIC	Clarithromycin	DEX, etoposide (1, UD)	0	-	UD
Sankhyan N, et al. (18)	India, 2014	Article	1/2	6–9 years	UD	UD	Shock (3), Seizures (2), Pneumonia (2)	UD	None	33.3% (1)	UD (the diagnosis of HLH was established only post mortem)	UD
He S, et al. (19)	China, 2014	Case	9/10	11 months-10 years	UD	4–22 days	Shock (1), MODS, Respiratory system, hepatic, hematologic alterations (5)	Chloramphenicol (15), Azithromycin (4)	mPLS (4, 3-7 days) DEX (10, 3-7 days)	21.1% (4)	MODS, Respiratory system, hepatic, hematologic alterations (4, ST was not diagnosed before death)	UD
Pazhaniyandi S, et al. (33)	India, 2015	Case	1/0	2 months	5 days	UD	Shock	Doxycycline	UD	0	-	UD
Zhou YH, et al. (34)	China, 2016	Case+Review	1/2	3–6 years	7–9 days (3)	UD	None	Chloramphenicol (3)	None	0	-	1 year
Jin Y, et al. (20)	China, 2016	Case	4/2	8 months-11 years	4–12 days	UD	ARDS (5), DIC (5), MOF (1)	Doxycycline (4), Azithromycin (2)	steroids (5, UD)	16.7% (1)	DIC, MOF	UD
Naoi T, et al. (21)	Japan, 2018	Review	7/6	2 month-11 years	4–10 days	9 days (2)	ARDS (7), Seizure (3),	Doxycycline (7), Azithromycin (2), Chloramphenicol (4)	DEX, cyclosporine, etoposide (1, UD), DEX, etoposide (1, UD), mPSL (5, UD)	7.7% (1)	UD	UD
Jin YM, et al. (6)	China, 2018	Article	6/10	1–12 years	2–14 days	UD	Shortness of breath (4), Cyanosis (2)	Azithromycin (6), Chloramphenicol (10)	DEX (2, 8-22 days)	6.3% (1)	MOF	UD
Agrwal S, et al. (22)	India, 2019	Case	1/0	8 months	10 days	15 days	Seizures	Doxycycline	DEX (1, 12 h)	100% (1)	Respiratory depression, pulmonary and gastric hemorrhage	/
Lin M, et al. (35)	China, 2019	Case	5/4	11 months-10 years	UD	UD	None	Chloramphenicol (7), Azithromycin (2)	DEX (7, UD)	0	-	UD
Basu A, et al. (17)	India, 2021	Article	11/7	1 month-12 years	UD	UD	ARDS (8) DIC (8) MODS (5) Myocarditis (2)	Doxycycline (18)	mPSL (4,UD)	11.1% (2)	UD	Undisclosed
Sahu SK, et al. (36)	India, 2021	Case	0/1	3 months	10 days	UD	Sepsis	Doxycycline	None	0	-	6.5 months
Zhang T, et al. (37)	China, 2021	Case	4/1	17–167 months	UD	UD	None	Doxycycline (5)	steroids (5, UD)	0	-	UD
Wu H, et al. (9)	China, 2022	Case	1/0	17 years	UD	UD	Respiratory failure, Septic shock, Blood coagulation dysfunction, Upper gastrointestinal bleeding, Cardiac insufficiency	Azithromycin (12 days), Doxycycline (UD)	DEX (1, 10 mg/day for 12 days and then tapered to 5 mg/day for 15 day)	0	-	UD
Fung RCM, et al. (38)	China, 2022	Case	1/0	7 years	7 days	UD	Shock, ARDS	Doxycycline	DEX (1, 10 mg/day for 2 weeks and then tapered to 5 mg/day for 1 week)	0	-	UD
Lu WM, et al. (23)	China, 2023	Article	9/6	(5.10 ± 3.82) years	UD	UD	ARDS (2)	Doxycycline (15)	Steroids (15, UD), etoposide (2, discontinued after diagnosis of ST)	6.7% (1)	UD	UD
Jian H, et al. (24)	China, 2024	Case	0/2	6 years, 10 years	9 days, 7 days	9 days, 7 days	Sepsis, pneumonia (2), Septic shock, acute kidney injury (1)	Doxycycline (2)	UD	0	-	UD

ARDS, acute respiratory distress syndrome; DIC, disseminated intravascular coagulation; MODS, multiple organ; dysfunction syndrome; DEX, dexamethasone; IVIG, intravenous immunoglobulin; mPSL, methylprednisolone; UD, undisclosed; MOF, multiple organ failure; CNS, central nervous system; ALF, acute liver failure; ARF, acute renal failure.

At present, mNGS can be used to diagnose ST in children, and ST can be easily treated; however, ST-associated HLH is often accompanied by other complications. Dyspnea (19 to 34%) may result from an excessive inflammatory response that induced pulmonary capillary leakage (27), and acute kidney injury (AKI) may develop from vascular inflammation leading to decreased renal blood flow and perfusion or vasculitis-induced serum albumin leakage (28). Hwang et al. (28) retrospectively analyzed the data of 510 patients with ST over 13 years (January 2001 to November 2013); they had a higher incidence of AKI (183, 35.9%) than that in the general population, but few of them required CRRT (of the 183 patients with ST and AKI, only 2 with chronic kidney disease were treated with CRRT). However, Bal et al. (29) showed that ST with AKI had a high mortality rate (20%, 9/45 cases). Once severe complications occur, it may be too late to recognize or ignore the potentially life-saving opportunities of following the HLH treatment protocol (6, 19, 20, 22). Therefore, professionals should be vigilant and should initiate HLH treatment protocols when ST-associated HLH occurs with other severe complications.

## Conclusion

Acute appendicitis may occur not only during the course of ST but also after successful treatment for ST. To date, this is the first reported case of acute appendicitis in a child who underwent laparoscopic appendectomy after being cured of ST. ST-associated HLH has a high probability of other complications and a high mortality rate when other severe complications are present. Pediatricians should be vigilant and should initiate HLH treatment protocols when ST-associated HLH occurs with other severe complications.

## Data Availability

The datasets presented in this study can be found in online repositories. The names of the repository/repositories and accession number(s) can be found in the article/supplementary material.

## References

[1] Elliott I Pearson I Dahal P Thomas NV Roberts T Newton PN. Scrub typhus ecology: a systematic review of Orientia in vectors and hosts. Parasit Vectors. (2019) 12:513. doi: 10.1186/s13071-019-3751-x, PMID: 31685019 PMC6829833

[2] Lamichhane P Pokhrel KM Alghalyini B Zaidi ARZ Alshehery MZ Khanal K. Epidemiology, clinical characteristics, diagnosis, and complications of scrub typhus infection in Nepal: a systematic review. Ann Med Surg. (2023) 85:5022–30. doi: 10.1097/MS9.0000000000001259, PMID: 37811079 PMC10553080

[3] Han L Zhang Y Jin X Ren H Teng Z Sun Z. Changing epidemiologic patterns of typhus group rickettsiosis and scrub typhus in China, 1950-2022. Int J Infect Dis. (2024) 140:52–61. doi: 10.1016/j.ijid.2023.12.013, PMID: 38163619

[4] Mukhopadhyay K Chakrabarty S Chatterjee C Misra SC. Mortality and complications of scrub typhus in the paediatric population: a systematic review and meta-analysis. Trans R Soc Trop Med Hyg. (2021) 115:1234–46. doi: 10.1093/trstmh/trab143, PMID: 34595519

[5] Asim M Amin F El-Menyar A. Multiple organ dysfunction syndrome: contemporary insights on the clinicopathological spectrum. Qatar Med J. (2020) 2020:22. doi: 10.5339/qmj.2020.22 33628712 PMC7884906

[6] Jin YM Liang DS Huang AR Zhou AH. Clinical characteristics and effective treatments of scrub typhus-associated hemophagocytic lymphohistiocytosis in children. J Adv Res. (2018) 15:111–6. doi: 10.1016/j.jare.2018.05.007, PMID: 30581619 PMC6300568

[7] Pathak S Chaudhary N Dhakal P Shakya D Dhungel P Neupane G. Clinical profile, complications and outcome of scrub typhus in children: a hospital based observational study in Central Nepal. PLoS One. (2019) 14:e0220905. doi: 10.1371/journal.pone.0220905, PMID: 31408484 PMC6692021

[8] Giri PP Roy J Saha A. Scrub typhus - a major cause of pediatric intensive care admission and multiple organ dysfunction syndrome: a single-center experience from India. Indian J Crit Care Med. (2018) 22:107–10. doi: 10.4103/ijccm.IJCCM_63_17, PMID: 29531452 PMC5842451

[9] Wu H Xiong X Zhu M Zhuo K Deng Y Cheng D. Successful diagnosis and treatment of scrub typhus associated with haemophagocytic lymphohistiocytosis and multiple organ dysfunction syndrome: a case report and literature review. Heliyon. (2022) 8:e11356. doi: 10.1016/j.heliyon.2022.e11356, PMID: 36411909 PMC9674499

[10] Xu G Walker DH Jupiter D Melby PC Arcari CM. A review of the global epidemiology of scrub typhus. PLoS Negl Trop Dis. (2017) 11:e0006062. doi: 10.1371/journal.pntd.0006062, PMID: 29099844 PMC5687757

[11] Musa TH Ahmad T Wana MN Li W Musa HH Sharun K. The epidemiology, diagnosis and management of scrub typhus disease in China. Hum Vaccin Immunother. (2021) 17:3795–805. doi: 10.1080/21645515.2021.1934355, PMID: 34124995 PMC8437466

[12] Zhang Y Zhang M Qin Y Zhang L Kang D Wei R. Epidemiological analysis and risk prediction of scrub typhus from 2006 to 2021 in Sichuan, China. Front Public Health. (2023) 11:1177578. doi: 10.3389/fpubh.2023.1177578, PMID: 37325301 PMC10261982

[13] Yang CH Young TG Peng MY Hsu GJ. Unusual presentation of acute abdomen in scrub typhus: a report of two cases. Zhonghua Yi Xue Za Zhi. (1995) 55:401–4. PMID: 7641127

[14] Lee CH Lee JH Yoon KJ Hwang JH Lee CS. Peritonitis in patients with scrub typhus. Am J Trop Med Hyg. (2012) 86:1046–8. doi: 10.4269/ajtmh.2012.11-0586, PMID: 22665616 PMC3366520

[15] Kundavaram AP Das S George VM. Scrub typhus presenting as an acute abdomen. J Glob Infect Dis. (2014) 6:17–8. doi: 10.4103/0974-777X.127943, PMID: 24741225 PMC3982349

[16] Rentea RM St Peter SD. Contemporary Management of Appendicitis in children. Adv Pediatr Infect Dis. (2017) 64:225–51. doi: 10.1016/j.yapd.2017.03.008, PMID: 28688590

[17] Basu A Chowdhoury SR Sarkar M Khemka A Mondal R Datta K. Scrub typhus-associated Hemophagocytic Lymphohistiocytosis: not a rare entity in pediatric age group. J Trop Pediatr. (2021) 67:fmab001. doi: 10.1093/tropej/fmab001, PMID: 33547467

[18] Sankhyan N Saptharishi LG Sasidaran K Kanga A Singhi SC. Clinical profile of scrub typhus in children and its association with hemophagocytic lymphohistiocytosis. Indian Pediatr. (2014) 51:651–3. doi: 10.1007/s13312-014-0470-4, PMID: 25129000

[19] He S Ge L Jin Y Huang A. Clinical analysis of scrub typhus-associated hemophagocytic syndrome. Zhonghua Er Ke Za Zhi. (2014) 52:683–7. doi: 10.3760/cma.j.issn.0578-1310.2014.09.009, PMID: 25476431

[20] Jin Y Huang L Fan H Lu G Xu Y Wu Z. Scrub typhus associated with hemophagocytic lymphohistiocytosis: a report of six pediatric patients. Exp Ther Med. (2016) 12:2729–34. doi: 10.3892/etm.2016.3668, PMID: 27698778 PMC5038170

[21] Naoi T Morita M Kawakami T Fujimoto S. Hemophagocytic Lymphohistiocytosis associated with scrub typhus: systematic review and comparison between pediatric and adult cases. Trop Med Infect Dis. (2018) 3:19. doi: 10.3390/tropicalmed3010019, PMID: 30274417 PMC6136620

[22] Agrwal S Dabas A Mantan M Yadav S. Hemophagocytic lymphohistiocytosis with neurological manifestations in an infant with scrub typhus: a rare fatal occurrence. Trop Dr. (2019) 49:52–3. doi: 10.1177/0049475518804696, PMID: 30360694

[23] Lu WM Yang XT Zhao MB Huang Y Xu L Jin HF. Analysis of clinical characteristics of scrub typhus associated with hemophagocytic syndrome in 15 children. China Trop Med. (2023) 23:255–9. doi: 10.13604/j.cnki.46-1064/r.2023.03.08

[24] Jian H Yang QX Duan JX Lai SY Che GL Teng J. mNGS helped diagnose scrub typhus-associated HLH in children: a report of two cases. Front Public Health. (2024) 12:1321123. doi: 10.3389/fpubh.2024.1321123, PMID: 38784570 PMC11111966

[25] Henter JI Horne A Aricó M Egeler RM Filipovich AH Imashuku S. HLH-2004: diagnostic and therapeutic guidelines for hemophagocytic lymphohistiocytosis. Pediatr Blood Cancer. (2007) 48:124–31. doi: 10.1002/pbc.21039, PMID: 16937360

[26] National Health Commission of the People’s Republic of China. (2019). Diagnosis and Treatment Guidelines for hemophagocytic syndrome in Children (2019 edition) [EB/OL]. Available online at: https://www.medsci.cn/guideline/show_article.do?id=7253c1c001e923ed (Accessed May 15, 2025).

[27] Narayanasamy DK Arun Babu T Vijayadevagaran V Kittu D Ananthakrishnan S. Predictors of severity in pediatric scrub typhus. Indian J Pediatr. (2018) 85:613–7. doi: 10.1007/s12098-018-2612-5, PMID: 29368107

[28] Hwang K Jang HN Lee TW Cho HS Bae E Chang SH. Incidence, risk factors and clinical outcomes of acute kidney injury associated with scrub typhus: a retrospective study of 510 consecutive patients in South Korea (2001-2013). BMJ Open. (2017) 7:e013882. doi: 10.1136/bmjopen-2016-013882, PMID: 28298367 PMC5353335

[29] Bal M Kar CR Behera HK Kar PC Biswas S Dixit S. Scrub typhus associated acute kidney injury: an emerging health problem in Odisha, India. J Vector Borne Dis. (2021) 58:359. doi: 10.4103/0972-9062.318318, PMID: 35381826

[30] Jayakrishnan MP Veny J Feroze M. Rickettsial infection with hemophagocytosis. Trop Dr. (2011) 41:111–2. doi: 10.1258/td.2010.100303, PMID: 21149571

[31] Han DK Baek HJ Shin MG Kim JW Kook H Hwang TJ. Scrub typhus-associated severe hemophagocytic lymphohistiocytosis with encephalomyelitis leading to permanent sequelae: a case report and review of the literature. J Pediatr Hematol Oncol. (2012) 34:531–3. doi: 10.1097/MPH.0b013e318257a442, PMID: 22627574

[32] Kwon HJ Yoo IH Lee JW Chung NG Cho B Kim HK. Life-threatening scrub typhus with hemophagocytosis and acute respiratory distress syndrome in an infant. J Trop Pediatr. (2013) 59:67–9. doi: 10.1093/tropej/fms030, PMID: 22735791

[33] Pazhaniyandi S Lenin R Sivathanu S. Hemophagocytic lymphohistiocytosis with a leukemoid reaction in an infant with scrub typhus. J Infect Public Health. (2015) 8:626–9. doi: 10.1016/j.jiph.2015.05.012, PMID: 26123173

[34] Zhou YH Xia FQ Van Poucke S Zheng MH. Successful treatment of scrub typhus-associated Hemophagocytic Lymphohistiocytosis with chloramphenicol: report of 3 pediatric cases and literature review. Medicine. (2016) 95:e2928. doi: 10.1097/MD.0000000000002928, PMID: 26937940 PMC4779037

[35] Lin M Huang A Zheng X Ge L He S. Misdiagnosis of scrub typhus complicated by hemophagocytic syndrome. BMC Pediatr. (2019) 19:102. doi: 10.1186/s12887-019-1475-x, PMID: 30971222 PMC6458710

[36] Sahu SK Behera JR Yadav SK. Scrub typhus with secondary hemophagocytic lymphohistiocytosis in a 3-month-old child from a tertiary care hospital of Odisha. Indian J Public Health. (2021) 65:85–6. doi: 10.4103/ijph.IJPH_565_20 33753698

[37] Zhang T Li X Zhou B Chen Y Tian J. A combination of doxycycline, IVIG, and glucocorticoids may be effective in the treatment of Hemophagocytic Lymphohistiocytosis secondary to Tsutsugamushi disease. J Pediatr Hematol Oncol. (2021) 43:e739–40. doi: 10.1097/MPH.0000000000002088, PMID: 33625088

[38] Fung RCM Leung KKY Au CC Cheong KN Kwan MYW Lam GKS. Paediatric acute respiratory distress syndrome and haemophagocytic lymphohistiocytosis complications of scrub typhus: a case report. Hong Kong Med J. (2022) 28:82–4. doi: 10.12809/hkmj208804, PMID: 35260498

